# Genotype-based prevalence of Birt-Hogg-Dubé syndrome in the healthcare and genomic registry populations – breaking the ‘rare disease’ status?

**DOI:** 10.1038/s41525-026-00563-2

**Published:** 2026-03-27

**Authors:** Izabela Broniarek, David J. Kwiatkowski, Neil Rajan, Kuniaki Seyama, Marcin Drzewiecki, Ireneusz Stolarek, Luiza Handschuh, Marek Figlerowicz, Piotr Kozlowski, Katarzyna Klonowska

**Affiliations:** 1 Department of Cancer Genetics, Institute of Bioorganic Chemistry, Polish Academy of Sciences, Poznan, Poland; 2 Cancer Genetics Laboratory, Division of Pulmonary and Critical Care Medicine, Brigham and Women’s Hospital, Harvard Medical School, Boston, MA USA; 3 Translational and Clinical Research Institute, Newcastle University, Newcastle upon Tyne, UK; 4 Department of Dermatology and NIHR Newcastle Biomedical Research Centre, Newcastle Hospitals NHS Foundation Trust, Newcastle upon Tyne, UK; 5 Division of Respiratory Medicine, Faculty of Medicine and Graduate School of Medicine, Juntendo University, Tokyo, Japan; 6 Department of Molecular and Systems Biology, Institute of Bioorganic Chemistry Polish Academy of Sciences, Poznan, Poland; 7 Laboratory of Genomics, Institute of Bioorganic Chemistry, Polish Academy of Sciences, Poznan, Poland; 8 Department of Molecular Genetics, Institute of Bioorganic Chemistry, Polish Academy of Sciences, Poznan, Poland

**Keywords:** Cancer, Diseases, Genetics, Medical research

## Abstract

The phenotype-based prevalence of Birt-Hogg-Dubé syndrome (BHD) is commonly estimated at 1 in 200,000–500,000. However, we demonstrate that BHD-causing *FLCN* variants are 75 to 180 times more prevalent in the multi-ethnic large genomic registry population. We highlight the urgent need for updated prevalence and penetrance estimates for BHD and other tumor suppressor gene syndromes, particularly among underrepresented non-European populations.

## Introduction

Birt-Hogg-Dubé syndrome (BHD) is a hereditary, autosomal dominant tumor suppressor gene syndrome. It is phenotypically heterogeneous, with tumor manifestations in multiple organs. Its hallmark clinical manifestations include benign skin tumors (fibrofoliculomas/trichodiscomas), lung cysts, spontaneous pneumothorax, and renal cell carcinoma^1^.

BHD is caused by loss-of-function variants in the tumor suppressor gene folliculin (*FLCN*), which is involved in numerous cellular processes, including tumor suppression, mitochondrial biogenesis, cell-cell adhesion, and autophagy^1^. Of note, *FLCN* is involved in the mechanistic target of rapamycin (mTOR) signaling pathway *via* regulation of mTORC1 and TFEB, which controls cell growth and metabolism in response to nutrient and energy levels.

BHD has been widely considered a rare hereditary disease (estimated prevalence of 1/200,000–500,000)^2,3^. However, it is thought to be underdiagnosed due to variable penetrance and a diverse range of clinical manifestations^4^.

## High frequency of deleterious *FLCN* variants in a large genomic registry population

Recently, Savatt et al.^5^ investigated the frequency of *FLCN* variants and the BHD clinical features in ~136,000 individuals from a healthcare system population, which led to the identification of a truncating *FLCN* variant in 1 per 3234 unrelated individuals. The study revealed that the BHD prevalence is 60 to 150 times higher than previously reported.

Intrigued by this finding, we investigated the frequency of deleterious *FLCN* variants using the Genome Aggregation Database (gnomAD) v4, which gathers 730,947 exomes and 76,215 genomes from unrelated individuals^6^. This is by far the biggest, well-curated genomic registry, which constitutes a powerful tool for estimating variant frequencies in populations of diverse genetic ancestries. Our gnomAD analysis focused exclusively on deleterious small variants (i.e., single-nucleotide variants and indels), as large deletions in *FLCN* are very rare. Only five nonrecurrent deletion events spanning one or more *FLCN* exons have been reported in the gnomAD v4 CNV and SV datasets.

**Fig. 1:**
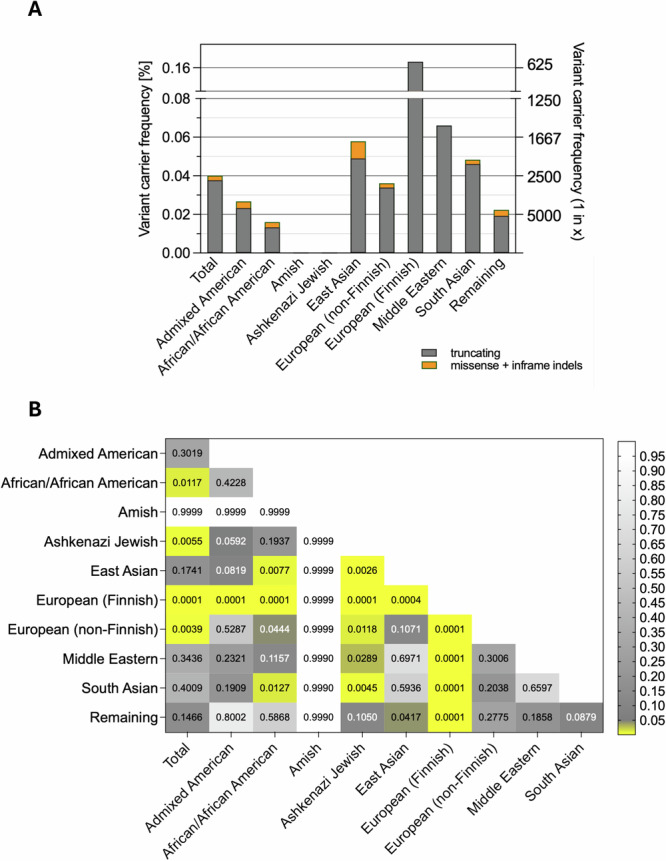
Frequencies of the deleterious *FLCN* variants. Data was extracted from the gnomAD v4.1. Truncating variants include nonsense, frameshift, and canonical splice donor/acceptor site variants. Inframe insertions/deletions and missense variants that are classified as pathogenic or likely pathogenic in ClinVar were included. **A** *FLCN* variant carrier frequency in different populations. **B** Differences in the deleterious *FLCN* variant frequencies in different populations. The P value was calculated using Fisher’s exact test. To calculate the P value, the data on the individuals from the given subpopulations was subtracted from the total population dataset. Therefore, depending on the subpopulation analyzed, the total population had different variant allele counts and total allele numbers. The color of boxes indicates statistical significance, as indicated in the legend on the right-hand side. Created with Graph Pad Prism 10.

Our analysis of the genomic registry population of mixed ethnicity (*n* = 807,162) revealed that the carrier frequency of truncating *FLCN* variants is 1 in 2654. The frequency is only slightly higher (1 in 2490) when all deleterious variants (i.e., pathogenic missense and inframe indel variants, in addition to truncating) are taken into account (Fig. 1A, Supplementary Data 1).

The *FLCN* variant frequency differs between populations (Fig. 1B). Notably, no variants were identified in the Ashkenazi Jewish population, in contrast to the Finnish population, where the deleterious *FLCN* variant frequency is strikingly high (1 in 614). There is also a marked difference in *FLCN* variant frequency in African/African American vs. other populations, including South and East Asian populations.

**Fig. 2:**
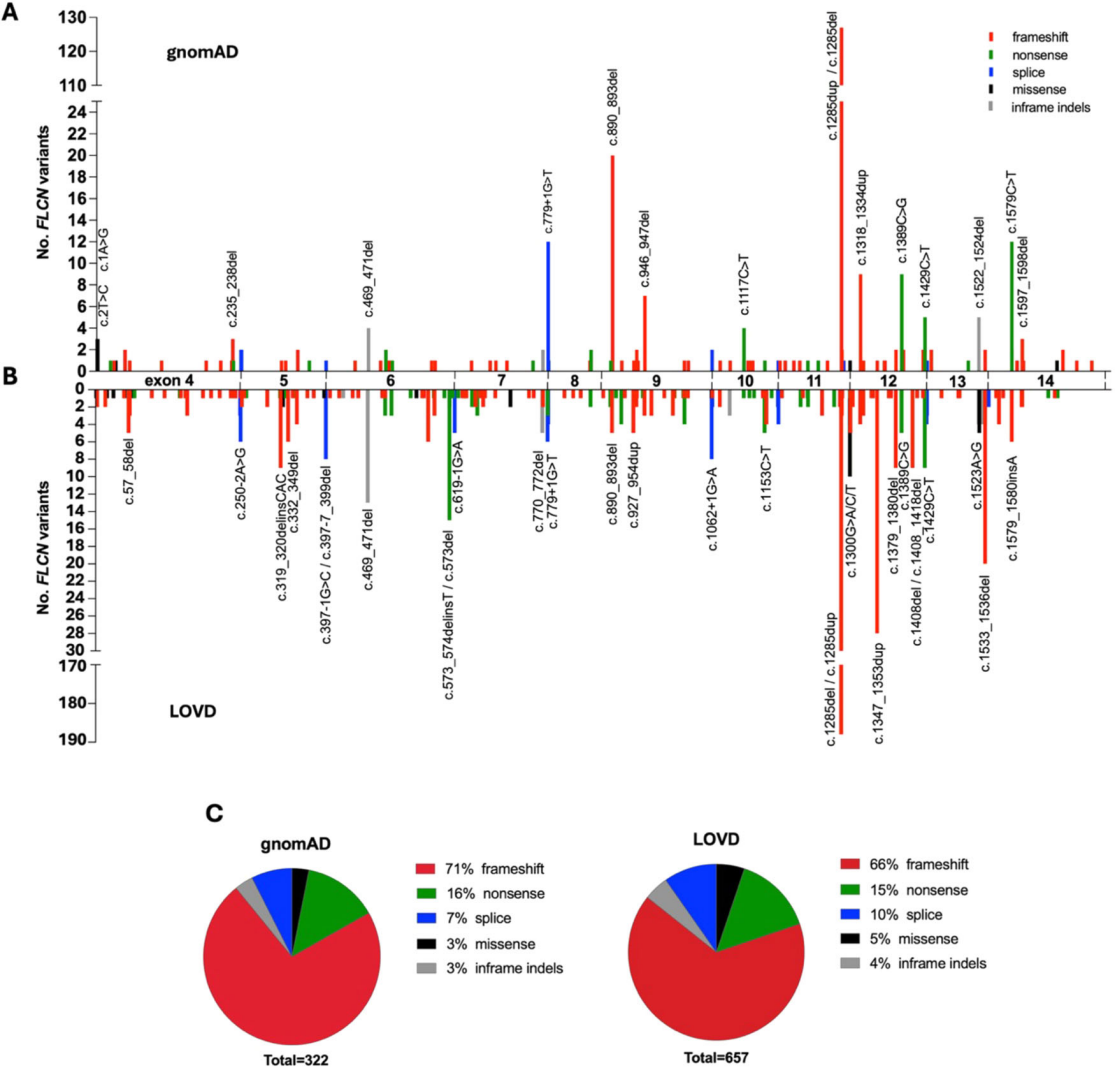
Maps of deleterious FLCN variants. **A, B** The y-axis indicates the number of *FLCN* variants along the *FLCN* sequence (x-axis). The color of the line indicates the variant type, as shown in the legend. Hotspot variants reported at least 3 times in gnomAD v.4.1 (**A**) or at least 5 times in LOVD v3.0 Build 29 (**B**) are specified on the graph according to HGVS nomenclature, based on ENST00000285071.9 reference sequence. **C** Pie charts showing proportions of the variant types reported in the databases. Created with Graph Pad Prism 10.

In total, we identified 98 unique deleterious variants in 322 carriers (Fig. 2A, Supplementary Data 2). The three most common types are frameshift (71%), nonsense (16%), and splice variants (7%) (Fig. 2C). The proportions of the variant types correspond well to the proportions of loss-of-function *FLCN* variants reported in Leiden Open Variation Database (LOVD)^7,8^ which is a well-curated resource gathering information about *FLCN* variants in BHD patients (Fig. 2B). This confirms the reliability of our gnomAD analysis results.

According to our analysis, the most common hotspot in *FLCN* is c.1285dup (Supplementary Data 2), occurring in 111 cases (32% of all deleterious variants). c.1285dup is a well-known hotspot reported frequently in BHD patients, including 142 times in LOVD. Other recurrent variants are c.1285del, c.890_893del, c.1579 C > T, and c.779+1 G > T, observed 46, 5, 1, and 6 times in LOVD, respectively. 10 out of 16 (63%) *FLCN* variants were observed recurrently (at least 3 times) in gnomAD.

Interestingly, 92% of all identified deleterious *FLCN* variants in the Finnish population are c.1285dup, which suggests that c.1285dup is a founder mutation in this population. We did not observe any other population-specific variant.

**Fig. 3:**
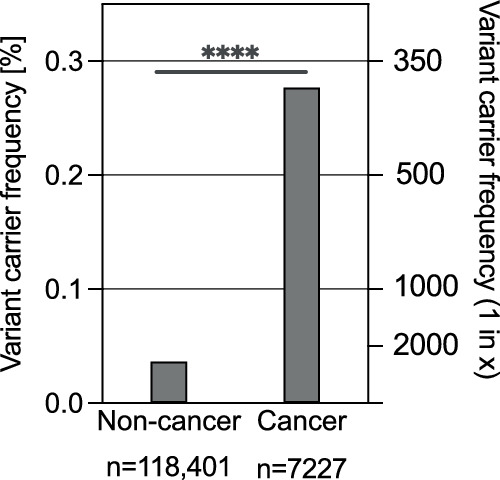
Frequencies of the deleterious *FLCN* variants in non-cancer and cancer populations. Data was extracted from the gnomAD v2.1. The *P*-value was calculated using Fisher’s exact test. Created with Graph Pad Prism 10.

Additionally, we performed an analogous analysis of data from gnomAD v2. This dataset is substantially smaller than gnomAD v4, but it enables comparison between non-cancer and cancer populations. Interestingly, the hotspot c.1285dup variant constituted 18 out of 20 (90%) *FLCN* variants identified in the cancer individuals, but a much smaller fraction (10 of 43, 23%) in non-cancer individuals. The overall carrier frequency was substantially higher for the cancer population (1/361) compared to the non-cancer population (1/2753) (*p* < 0.0001) (Fig. 3, Supplementary Data 3), suggesting an association between *FLCN* variants and cancer predisposition. Although this association may be partially explained by the occurrence of renal cell carcinoma, which falls within the spectrum of BHD-related clinical manifestations, it is likely that the risk of other cancers is also increased among *FLCN* mutation carriers. However, this cannot be examined due to a lack of information about cancer type in the gnomAD cohort. The association of the *FLCN* variants with cancer predisposition warrants further investigation in the future.

For an independent comparison, we analyzed the *FLCN* genotypes in the genomes of individuals from the Polish population, sequenced as part of the Genomic Map of Poland Project^9^. To the best of our knowledge, this is the largest and the only genomic registry dataset available for the Polish population. None of the 4968 individuals had a deleterious *FLCN* variant. This result does not allow for the calculation of an exact frequency of deleterious *FLCN* variants in the Polish population; however, it can be concluded that the frequency is likely not significantly higher than in the gnomAD European non-Finnish population.

## Discrepancies in disease prevalence based on clinical phenotype vs. genotype assessment

Our analyses performed in the genomic registry population are in agreement with the recent report of Yngvadottir et al.^10^. Using data from the UK Biobank and the 100,000 Genomes Project, they analyzed the genomes of over 500,000 individuals (primarily of European ancestry) and demonstrated that the frequency of truncating *FLCN* variants is 1 in 2710 (100,000 Genomes Project) to 1 in 4190 (UK Biobank).

Our results are in line with studies by Savatt et al.^5^ and van Riel et al.^11^, which were performed using healthcare system data; the studies have consistently demonstrated that, in prior reports, the frequency of truncating *FLCN* variant carriers and BHD prevalence were strongly underestimated.

These observations are in agreement with other reports on discrepancies between disease prevalences estimated based on clinical phenotype vs. genotype assessment in large-scale population genetic studies. Interestingly, it also concerns disorders with a relatively severe phenotype, which seems to be hard to overlook in daily life and/or during consultation with expert clinicians. For instance, using genome sequencing data from 82,176 participants and modeling disease prevalence, Ibanez et al.^12^ showed that the expected number of individuals with neurological repeat expansion disorders is two to three times higher than currently reported. Similar conclusions on hereditary thrombotic thrombocytopenic purpura and von Willebrand disease can be drawn from studies by Seidizadeh et al.^13,14^. Based on data from gnomAD v4, disease-causing genotypes are considerably more prevalent than the currently reported incidence of both diseases. Similarly, a recent study of neurofibromatosis type 1 (NF1) by Safonov et al.^15^ revealed that the prevalence of disease-causing genotypes is more than twice as high as the phenotype-based prevalence of NF1. Half of the individuals with a pathogenic variant in this study lack the classic NF1 clinical manifestations, with many carriers of a post-zygotic mosaic variant. In addition, incidental pathogenic findings are associated with an increased risk of malignancy. This valuable finding shows that individuals with incidental pathogenic variants without an expected clinical phenotype require specialized medical management.

The above-mentioned reports and others^16,17^ highlight the need for updated estimates of disease prevalence to better understand the risk of genetic disorders. It is of high importance, especially to non-European populations, who are underrepresented in sequence databases.

## Underestimation of the Birt-Hogg-Dubé syndrome prevalence: underdiagnosis or low penetrance?

Findings of Savatt et al. suggest that many carriers of *FLCN* pathogenic variants may remain undiagnosed. A BHD diagnosis can be challenging because the manifestations of BHD are heterogeneous and can mimic those of other disorders. Differential diagnosis is important, especially for fibrofoliculomas/trichodiscomas skin lesions, which are the most common manifestations leading to the diagnosis of BHD^18^. There are at least two skin conditions that may be mistaken for BHD: familial multiple discoid fibromas^19,20^ and a novel disorder caused by a pathogenic variant in the *PRDM10* gene^21^.

Underdiagnosis of BHD does not appear to be the only reason for the discrepancy between the prevalence of pathogenic *FLCN* variants and the number of diagnosed patients. This may be related to a lower disease penetrance than previously reported. When it comes to the inherited, organ-specific diseases, the available clinical data are often sufficient for accurate penetrance estimation. An example of such analysis is an estimation of cardiomyopathy penetrance performed by Figueiral et al.^22^, which revealed high penetrance of the disease among individuals identified as carriers of pathogenic variants in cardiomyopathy genes in a biobank population database. The penetrance of an inherited multisystemic disorder like BHD is more difficult to determine. Previous BHD penetrance estimates were based mainly on data from BHD families with well-documented cases^23,24^. To date, only two studies have attempted to determine the risk of BHD manifestation in carriers of pathogenic *FLCN* variants using data from large database cohorts of unrelated individuals^5,10^. Analysis of data from a biobank (UK Biobank) and a healthcare system revealed that the frequency of pneumothorax and renal cell carcinoma is lower in individuals without a family history of BHD. The penetrance of BHD requires further investigation, particularly regarding skin manifestations and lung cysts. Determining the modifiers of BHD penetrance is a fundamental step toward understanding the pathology of this disorder.

With the growing volume of genome sequencing data, pathogenic variants are being increasingly identified as incidental findings in individuals without any clinically recognized manifestations or a family history of the disease. Because the age of onset, progression, and development of the disease can differ greatly between individuals with and without a family history of the disease, the development of effective programs enabling follow-up of the health status of individuals with incidental findings is of high importance in the context of tumor suppressor gene syndromes and other genetic disorders.

This study has several limitations. The sample sizes for some populations may not be large enough to assess variants occurring at the expected frequencies ranging from 1 in 2,000 to 1 in 4,000. As a result, accurately estimating the frequency of pathogenic *FLCN* variants in the Middle Eastern (gnomAD), Amish (gnomAD), and Polish (the Genomic Map of Poland) populations is difficult and may be biased. It is also worth noting that gnomAD v4 gathers data from various large-scale sequencing projects, including data from biobank and disease-focused cohorts. The disease-focused projects contributing to gnomAD v4 mainly regard cardiovascular disease, mental disorders, Alzheimer’s disease, type 2 diabetes, and inflammatory bowel diseases. As a result, gnomAD v4 includes data from control (14% of all exomes) and disease (17% of all exomes) cohorts, as well as a large fraction of data from individuals with unknown phenotype (69% of all exomes). Therefore, data gathered in gnomAD may reflect ascertainment, recruitment, and ancestry biases, and the gnomAD allele frequencies should not be interpreted as direct estimates of prevalence in the general population.

Ethical approval was not required for the analysis of data from gnomAD, as it is an open-source database. The analyses performed as part of the Genomic Map of Poland Project were approved by the Bioethics Committee at the District Medical Chamber in Gdansk, Poland (KB - 30/20) and the Bioethics Committee at the Karol Marcinkowski Medical University in Poznan, Poland (630/20).

## Supplementary information

Supplementary Information

Supplementary Data.

## Data Availability

The gnomAD datasets analyzed in this study are available in the gnomAD database, https://gnomad.broadinstitute.org/. The datasets generated by the Genomic Map of Poland Project and analyzed during the current study are available from the corresponding author on reasonable request.

## References

[1] Schmidt, L. S. & Linehan, W. M. Molecular genetics and clinical features of Birt–Hogg–Dubé syndrome. *Nat. Rev. Urol.* **12**, 558–569 (2015). 26334087 10.1038/nrurol.2015.206 PMC5119524

[2] Muller, M. E., Daccord, C., Taffé, P. & Lazor, R. Prevalence of Birt-Hogg-Dubé Syndrome Determined Through Epidemiological Data on Spontaneous Pneumothorax and Bayes Theorem. *Front. Med*. *(**Lausanne**)*. **8**, 631168 (2021). 10.3389/fmed.2021.631168 PMC8111214 33987191

[3] Sattler, E. & Steinlein, O. Birt-Hogg-Dubé syndrome. https://www.orpha.net/en/disease/detail/122?name=Birt-Hogg-Dub%C3%A9+syndrome&mode=name (2023). 10.3389/fmed.2023.1289948 PMC10663224 38020174

[4] Woodford, M. R. et al. Seventh BHD International Symposium: Recent Scientific and Clinical Advancement. *Oncotarget* **13**, www.oncotarget.com (2022). 10.18632/oncotarget.28176 PMC8780807 35070081

[5] Savatt, J. M. et al. Frequency of truncating FLCN variants and Birt-Hogg-Dubé–associated phenotypes in a health care system population. *Genet. Med.* **24**, 1857–1866 (2022). 35639097 10.1016/j.gim.2022.05.006 PMC9703446

[6] Gudmundsson, S. et al. Exploring penetrance of clinically relevant variants in over 800,000 humans from the Genome Aggregation Database. *Nat. Commun.* **16**, 9623 (2025). 41173899 10.1038/s41467-025-61698-x PMC12579199

[7] Fokkema, I. F. A. C. et al. The LOVD3 platform: efficient genome-wide sharing of genetic variants. *Eur. J. Hum. Genet.* **29**, 1796–1803 (2021). 34521998 10.1038/s41431-021-00959-x PMC8632977

[8] Lim, D. H. K. et al. A new Locus-Specific Database (LSDB) for mutations in the folliculin (FLCN) gene. *Hum. Mutat.* **31**, E1043–E1051 (2010). 19802896 10.1002/humu.21130

[9] Genomic Map of Poland – European Center for Bioinformatics and Genomics. https://genompolski.pl/.

[10] Yngvadottir, B. et al. Inherited predisposition to pneumothorax: estimating the frequency of Birt-Hogg-Dubé syndrome from genomics and population cohorts. *Thorax* **80**, 553–555 (2025). 40210444 10.1136/thorax-2024-221738 PMC12322431

[11] van Riel, L. et al. Correspondence on “Frequency of truncating FLCN variants and Birt-Hogg-Dubé-associated phenotypes in a health care system population” by Savatt et al. *Genet. Med.* **25**, 158–160 (2023). 36383210 10.1016/j.gim.2022.08.033

[12] Ibañez, K. et al. Increased frequency of repeat expansion mutations across different populations. *Nat. Med.* **30**, 3357–3368 (2024). 39354197 10.1038/s41591-024-03190-5 PMC11564083

[13] Seidizadeh, O., Cairo, A., Baronciani, L., Valenti, L. & Peyvandi, F. Population-based prevalence and mutational landscape of von Willebrand disease using large-scale genetic databases. *NPJ Genom. Med.* **8**, 31 (2023). 37845247 10.1038/s41525-023-00375-8 PMC10579253

[14] Seidizadeh, O., Cairo, A., Mancini, I., George, J. N. & Peyvandi, F. Global prevalence of hereditary thrombotic thrombocytopenic purpura determined by genetic analysis. *Blood Adv.* **8**, 4386–4396 (2024). 38935915 10.1182/bloodadvances.2024013421 PMC11375255

[15] Safonov, A. et al. A genotype-first approach identifies high incidence of NF1 pathogenic variants with distinct disease associations. *Nat. Commun.* **16**, 3121 (2025). 40169570 10.1038/s41467-025-57077-1 PMC11962086

[16] Schmitz, M. J. et al. Leveraging diverse genomic data to guide equitable carrier screening: Insights from gnomAD v.4.1.0. *Am. J. Hum. Genet.* **112**, 181–195 (2025). 39615480 10.1016/j.ajhg.2024.11.004 PMC11739870

[17] Sun, K. Y. et al. A deep catalogue of protein-coding variation in 983,578 individuals. *Nature* **631**, 583–592 (2024). 38768635 10.1038/s41586-024-07556-0 PMC11254753

[18] Lim, D. H. K. The Clinical and Molecular Genetic Investigation of Genetic Conditions Predisposing to Kidney Cancers. (University of Birmingham, 2016).

[19] van de Beek, I. et al. Familial multiple discoid fibromas is linked to a locus on chromosome 5 including the FNIP1 gene. *J. Hum. Genet.* **68**, 273–279 (2023). 36599954 10.1038/s10038-022-01113-1

[20] Starink, T. M. et al. Familial multiple discoid fibromas: A look-alike of Birt-Hogg-Dubé syndrome not linked to the FLCN locus. *J. Am. Acad. Dermatol.* **66**, 259.e1–259.e9 (2012). 21794948 10.1016/j.jaad.2010.11.039

[21] van de Beek, I. et al. PRDM10 directs FLCN expression in a novel disorder overlapping with Birt–Hogg–Dubé syndrome and familial lipomatosis. *Hum. Mol. Genet.* **32**, 1223–1235 (2023). 36440963 10.1093/hmg/ddac288 PMC10026250

[22] Figueiral, M. et al. Prevalence, Penetrance, and Phenotypic Manifestation of Cardiomyopathy-Associated Genetic Variants in the General Population: Insights from a Mayo Clinic Biobank Study. *Mayo Clin. Proc.* **99**, 1732–1743 (2024). 39387793 10.1016/j.mayocp.2024.05.027

[23] Matsumoto, K., Lim, D., Pharoah, P. D., Maher, E. R. & Marciniak, S. J. A systematic review assessing the existence of pneumothorax-only variants of FLCN. Implications for lifelong surveillance of renal tumours. *Eur. J. Hum. Genet.* **29**, 1595–1600 (2021). 34267338 10.1038/s41431-021-00921-x PMC8560836

[24] Bruinsma, F. J. et al. Update of penetrance estimates in Birt-Hogg-Dubé syndrome. *J. Med. Genet.* **60**, 317–326 (2023). 36849229 10.1136/jmg-2022-109104

