# Supplementary material for: Genotype-based prevalence of Birt-Hogg-Dubé syndrome in the healthcare and genomic registry populations – breaking the ‘rare disease’ status?
Source: NPJ Genom Med. 2026 Mar 27;11:17. doi: 10.1038/s41525-026-00563-2 (PMC13031786; doi:10.1038/s41525-026-00563-2)
Supplement: Supplementary file 1 — Supplementary Information [file 41525_2026_563_MOESM1_ESM.pdf]

Supplementary Information

Supplementary Data 1 - Frequencies of deleterious *FLCN* variants in different populations. (xlsx)

Supplementary Data 2 – List of deleterious *FLCN* variants reported in gnomAD v4 (xlsx)

Supplementary Data 3 – Lists of deleterious *FLCN* variants reported in gnomAD v2 in non-cancer and cancer populations (xlsx)
